# Targeting NLRP3 and AIM2 signaling pathways by Viscosol alleviates metabolic dysregulations induced inflammatory responses in diabetic neuro- and nephropathy: An *in silico* and *in vivo* study

**DOI:** 10.1371/journal.pone.0313816

**Published:** 2025-04-02

**Authors:** Summan Thahiem, Muhammad Ihsan, Hamza Muneer, Aamir Sohail, Mehmand Khan, Iram Murtaza, Zia Uddin, Muhammad Shafique, Khalid J. Alzahrani, Hamid Ali, Imran Ullah

**Affiliations:** 1 Department of Biochemistry, Faculty of Biological Sciences, Quaid-i-Azam University, Islamabad, Pakistan; 2 Department of Biosciences, COMSATS University Islamabad, Tarlai Kalan, Islamabad, Pakistan; 3 Department of Biochemistry & Biotechnology, MNS University of Agriculture Multan, Multan, Pakistan; 4 Department of Pharmacy, COMSATS University Islamabad, Abbottabad, KP, Pakistan; 5 Department of Pharmacology, College of Pharmacy, Shaqra University, Shaqra, Saudi Arabia; 6 Department of Clinical Laboratories Sciences, College of Applied Medical Sciences, Taif University, Taif, Saudi Arabia; GLA University, India

## Abstract

Type 2 Diabetes (T2D) is a chronic metabolic disorder, considered the fastest growing pandemic of the 21^st^century. Meta-inflammation is a pivotal characteristic of T2D. Hyperactivated PTP1B, NLRP3, and AIM2 inflammasomes are considered the major regulators of metabolic inflammation. The concept of diabetes as an inflammatory disease has changed the pathogenic vision of T2D and hence, the compounds that mitigateinflammation in the setting of T2D are under the limelight of research. Current study aimed to evaluatethe anti-inflammatory potency of Viscosol, a novel PTP1B inhibitor, isolated from *Dodonaea viscosa*, in the STZ-HFD-induced T2D mouse model. Herein, male mice(C57BL/6), were administrated with Streptozotocin (STZ) (40mg/kg) and Viscosol (33mg/kg), intraperitoneally. Computational profiling revealed good absorption, distribution, metabolism and excretion (ADME) properties, least toxicity, and high docking score of Viscosol with PTP1B(−6.4 kcal/mol), NLRP3(−7.2 kcal/mol), and AIM2(−7.4 kcal/mol). Viscosol treatment significantly restored normal body weight (*p* < 0.0001), decreased the blood glucose level (*p* < 0.001), serum ROS level(*p* < 0.05) and diminished the severity of histopathological lesions, inflammatory lobules and increased the cell count of both brain and kidney tissues. The RT-qPCR analysis showed that Viscosol significantly reduced the mRNA expression of *PTP1B, NF-κB, NLRP3*, and *AIM2*up to 2.7-folds, 2.6-folds, 5.7-folds and 14.2-folds in the kidney tissues and 1.6-folds, 1.2-folds, 10.2-folds and 1.5-folds in brain tissues. Conclusively, inhibition of PTP1B via Viscosol could attenuate meta-inflammation by suppressing the aberrant NLRP3 and AIM2 inflammasome signaling in diabetes-linked pathophysiology.

## Introduction

Type 2 Diabetes (T2D) is a chronic metabolic disease, manifested by a wide range of pathologies including hyperglycemia, hyperlipidemia, oxidative stress, and low-grade inflammation [1]. According to the International Diabetes Federation (IDF) 2021 report, approx. 537 million people are suffering from diabetes worldwide [2]. Various studies implicated that diabetes induced metabolic and hemodynamic dysfunctions augmented the reactive oxygen species (ROS) levels which lead to the increased production of inflammatory cytokines [3]. It is evident that oxidative stress plays a key role in the pathogenesis and progression of sustained inflammatory state in T2D [4].

Recent evidence implies a bidirectional link between inflammation and metabolism by suggesting that metabolic inflammation is a pivotal characteristic of T2D [5].The manifestation of this proinflammatory state includes hyperactivated innate immune responses, in which inflammasome complexes are the integral components. They function as unique sensors for metabolic dysregulation. NLRFamily, PyrinDomain Containing3(NLRP3) and Absent in Melanoma 2 (AIM2) inflammasomes are best known so far for their involvement in T2D [6,7].Several metabolic DAMPs such as (ROS, disrupted lysosomal trafficking, aberrant ionicflux, and cholesterol crystals)lead to assembly of inflammasome components, i.e., NLRP3/AIM2, apoptosis-associated speck-like protein containing a CARD (ASC), and caspase-1,subsequentlyreleasing inflammatory cytokinesIL-1*β* and IL-18,andamplifying the inflammatory cascade [8]. It is worth emphasizing that inflammatory mediatorsIL-6 and Tumor Necrosis Factor α (TNF-α), lead to the activation of Protein Tyrosine Phosphatase 1B (PTP1B) [9,10]. It hydrolyzes the tyrosine phosphorylation of Insulin Receptor (INSR) and Insulin Receptor Substrate-1(IRS-1),thus acting as a negative regulator of insulin signaling pathway [11,12]. PTP1B is also involved in the modulation of inflammatory mechanisms by regulating Nuclear Factor Kappa B (NF-κB) [13]. These properties renderPTP1B an attractive target for therapeutic interventions.

Viscosol (5,7-dihydroxy-3,6- dimethoxy-2-(4-methoxy-3-(3- methyl but- 2-enyl) phenyl)-4H-chromen-4-one) isolated from *Dodonaeaviscosa* is a metabolically active naturally occurring prenylated flavonoid and potent PTP1B inhibitor [14]. The antidiabetic potential of Viscosol has already been confirmed *in vivo* in our previous study [15], and now we aimed to investigate its pharmacological profile, antioxidant, and anti-inflammatory potential through *in silico* and *in vivo* studies. Pharmacokinetic, BOILED-Egg model, and ADME/T parameters of Viscosol were evaluated via computational tools. Molecular docking was performed to investigate the active Viscosol’s interactions, binding affinity, and inhibitor binding manner with PTP1B, NLRP3, and AIM2. Furthermore, we investigated the putative association between the intricate molecular mechanisms linking PTP1B, NLRP3 and AIM2 inflammasomes as well as elucidatingtheanti-inflammatory potency of Viscosol*in vivo*.

## Materials and methods

### Materials

Streptozotocin(STZ)waspurchasedfrom (Bioworld, Catalog No. # 41910012-3). Invitrogen RNA kit (PureLink TM RNA Minikit) was purchased from Thermofisher Scientific, Cat No.# 1218301 8A).cDNA synthesis kit (Thermo scientific Revert Aid First Strand) was purchased from Thermofisher scientific. Invitrogen TRIzolreagent was purchased from Thermofisher Scientific. SYBR Green (Thermo Scientific Maxima SYBR Green/ROX qPCR Master Mix (2x) was purchased from Thermofisher Scientific. Viscosol was sourced from Dr. Ziauddin, Assistant professor, at COMSATS University, who previously isolated this compound from aerial parts of *Dodonaeaviscosa*.

### Ethical approval and in vivo experimental design

To investigate the anti-inflammatory activity of Viscosol *in vivo*, we developed a low-dose streptozotocin (STZ) and High Fat Diet (HFD)-induced mouse model. All mice received humane care and the entire experiment was conducted according to the guidelines approved by the Institutional Review Board of COMSATS University, Quaid-i-Azam University and National Institute of Health (NIH guidelines Islamabad, Pakistan)as per the considerations of the U.K. Animals (Scientific Procedures) Act, 1986.All experimental animals were euthanized via cervical dislocation method by trained personnels of animal house facility of Quaid-e-Azam University, to ensure least painful death according to American Veterinary Medical Association (AVMA) guidelines [16].

### Dose regimenforT2D model development

Male mice(Mus musculus, strain C57BL/6), weighing 25–40 g (8–12 weeks old) were selected as an experimental model. Mice were acquired from the NIH, Islamabad primate facility, and acclimatized for 1 week under standard conditions (27 °C with 12 hours of light & dark cycles). The T2D mouse model was developed by following our modified protocol [15].Animals were randomly divided into three groups, each containing 3 mice. In Group 1 (Control), mice were provided with standard feed and water. On day 11, saline solution was injected intraperitoneally at the fasting state. In group 2 (HFD-STZ-induced diabetic group) and group 3 (STZ-HFD Viscosol-treated) mice, diabetes was induced by administering STZ injection consecutively for 5 days at 4–6 hours of fasting. Mice having fasting BGL > 250 mg/dL were considered diabetic. In group 3, after confirmation of diabetes on day 11,Viscosol was injected intraperitoneally at 4–6 hours of fasting state. At the end of the experiment (day 17), mice were euthanized and dissected for further studies. Organs were extracted for morphological as well as molecular studies. All experiments were performed in triplicate.

### Baseline parameters assessment

Persistent hyperglycemia and abrupt weight loss are the main hallmarks of T2D. For confirmation of T2D in our model, the body weight and blood glucose level (BGL) of all mice were monitored throughout the experimental timeline.

### Oxidative stress profiling using D-ROM assay

The serum ROS profile of all the groups was estimated by a high-throughput, and cost-effective D-ROM assay by following previously mentioned protocol [17]. We have used two reagents (1 mg/1 ml) termed as reagent 1 (N, N-diethyl para-phenyl diamine sulphate) a chromogen and reagent 2 (0.5% FeSO4) and sodium acetate buffer (NaCH3COO) having a pH of 4.8, and molarity of 0.1M. Both reagents (R1 and R2) were mixed well, maintaining the ratio of 1:24 to set up ahomogeneousreactionmixture. Absorbance was checked at 505nm by spectrophotometer (Multiskan GO, Thermo Fischer Scientific, USA).

### H & E staining and quantification

The effect of Viscosol was further elucidated by histological examination using Hematoxylin and Eosin (H&E) staining. Tissue sections were separated and fixed in 10% formalin to protect them from subsequent processing and intramolecular changes and then stored at room temperature. The tissue sections were stained by Hematoxylin and Eosin dyes and examined at 10X magnification, under the bright field microscope (CX41, Olympus Microscope, Japan). Furthermore, Image J software was used for quantitative analysis of histological damage and inflammatory lobules.

### RNA extraction and cDNA synthesis

We used Invitrogen RNA kit protocol to extract the RNA. Quantification of extracted RNA was done by using the Nanodrop Spectrophotometer (Berthold Detection System GmbH 75173 Pforzheim, Germany). cDNA was synthesized from purified RNA using a high-capacity cDNA synthesis kit (Thermo scientific Revert Aid First Strand). After preparing the reaction mixture, the tubes were incubated for 1 hour in a PCR machine (T3 Thermoblock, Biometra, Germany) at 37 °C.

### RT-qPCR

Expression analysis of targeted genes was performed by real-time PCR (MyGo PCR systems, IT-IS life sciences) by using SYBR Green (Thermo Scientific Maxima SYBR Green/ROX qPCR Master Mix (2x)). The list of primers is shown in the supplementary file (S1 Table ). For normalization, *HPRT1* and *PPiA*were used as an internal control for kidney and brain, respectively. All reactions were run in triplicate to account for any run-to-run variability.

### *In silico* ADMET profiling of Viscosol

Pharmacokinetic features, physiochemical properties as well as ADME/T assessment of novel compounds via computational and machine learning tools is a crucial aspect to consider in drug discovery. It predicts drug efficacy, mode of administration, pharmacological profile, and biosafety before *in vivo* experimentation [18]. ADME parameters of Viscosol were determined by ‘SwissADME’ software(http://www.swissadme.ch/index.php) using canonical SMILES retrieved from PubChem(https://pubchem.ncbi.nlm.nih.gov/). Viscosol was further screened using Lipinski’s, Egan, Ghose, and Veber’s filters, along with Brain or Intestinal Estimated permeation (BOILED-egg) model. The bioavailability score was calculated through the online web tool ‘MolSoft’ (https://molsoft.com/mprop/) and Viscosol’s toxicity was estimated through ‘ProTox-II’ tool (https://tox-new.charite.de/protox_II/).

### Molecular docking studies on PTP1B, NLRP3 and AIM2

#### Proteins and ligandspreparation.

The 3D structure of Viscosol was retrieved from PubChem Database(https://pubchem.ncbi.nlm.nih.gov/). The crystallographic structures of PTP1B (PDB ID: 1NNY), NLRP3 (PDB ID: 7ALV), and AIM2 (PDB ID: 3RN2) along with their inhibitors were obtained from RCSB, Protein Data Bank (https://www.rcsb.org/) [19]. The miscellaneous ligands and water molecules were removed for further docking simulations by Discovery Studio Visualizer 16.1 (Accelrys, Inc., San Diego, CA, USA). The 2D and 3D structures of Viscosol and standard drug Ibuprofen were constructed using Chemdraw 2D (Chemdraw professional 19, Spartan’20, Wavefunction Inc., CA, USA).

#### Molecular docking.

We further carried out molecular docking studies to comprehend the interaction and binding pattern of the Viscosol with PTP1B, NLRP3, and AIM2, using AutoDock 4.2 [20]and PyRx(https://pyrx.sourceforge.io/) [21].The results were visualized and evaluatedusing Discovery StudioVisualizer [22].

### Statistical analysis

The RT-qPCR data was analyzed by the 2^ − ΔΔCT^ method to evaluate the relative fold change expression of genes. Graph Pad Prism (version 9.3.0) was used for descriptive statistics to apply one-way ANOVA followed by Tukey’s test. Histological microphotographs were processed and analyzed by Image J software. The means of all groups were described as mean ±  SD. Results were considered significant at a *p-*value ≤  0.05. All experiments were run in triplicate.

## Results

### Pharmacodynamicsanalysis

**Fig 1:**
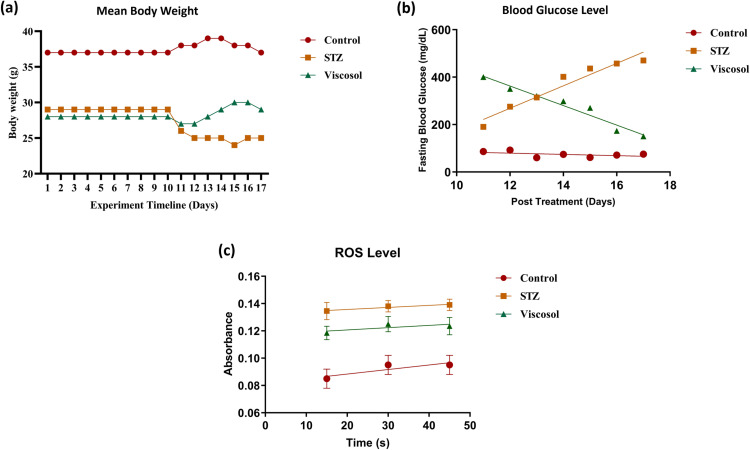
Baseline characteristics and oxidative profiling. (a) Mean body weight (g) of all mice against experimental days. A significant regain in body weight was observed in the Viscosol treated group(****p < 0.0001). (b) Blood glucose profiling. The graphshowsadecrease in blood glucose level in the presence of Viscosol (***p < 0.001). (c) ROS profiling.Viscosol treatment significantly reduced the ROS level (*p < 0.05).

Our model mimicked the pathophysiological features of T2D, i.e., dramatic weight reduction and escalated serum BGL. Mice body weight and BGL was monitored throughout the treatment days (11^th^ to 17^th^ day) in all three groups at fasting state. In Viscosol treated group, a significant regain in body weight was observed (****p < 0.0001)as shown in(Fig 1a).Furthermore, Viscosol treatment significantly decreased the BGL of all mice (***p < 0.001) as shown in(Fig 1b).

### Viscosolabolisheddiabetes-induced oxidative stress

In T2D, oxidative stress is the main culprit behind post-diabetic inflammation. Hence, we investigated the blood oxidative profile. In the Viscosol treated group, there was a significantabrogation in serum ROS level as compared to the STZgroup(***p < 0.0531). Therefore, suggesting the antioxidant properties of Viscosol. Details are illustrated in (Fig 1c).

### Protective effect of Viscosol on nephro- and neuro histopathology

**Fig 2:**
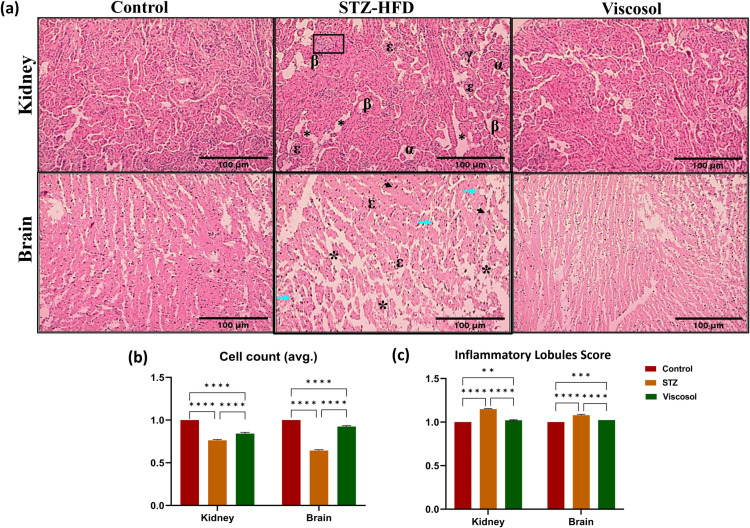
Photomicrographs of H & E-stained kidney and brain section at 10X. (a) Normal morphology of the kidney tissue; STZ group, presenting histological changes; tubular dilation (*), epithelial cell necrosis (α), degeneration of renal tubules (β), tubular interstitial fibrosis (box), glomeruli shrinkage and thickening of bowman capsule (ε) mesangial matrix deposition (γ); partial retrieval towards normal morphology in Viscosol treated group. The second panel shows brain normal tissues; HFD-STZ group, presenting altered tissue morphology having increased axonal swelling (arrowhead), loss of axons (neon arrows), lost myelin sheath (ε), loss of Schwann cells (arrow), and neuronal disorganization (*); Viscosol treated group, showing partial retrieval towards normal morphology. (b) Cell count score wassignificantly higher in Viscosol treated group as compared to STZ group [Kidney: STZ =  0.76 ±  0.01, Viscosol =  0.84 ±  0.015, ****p < 0.0001); (Brain: STZ =  0.64 ±  0.01, Viscosol =  0.924 ±  0.01, ****p < 0.0001]. (c) Inflammatory lobules score showed significant reduction in inflammatory lobules in the Viscosol treated grou*p* as compared to STZ group [Kidney: STZ =  1.15 ±  0.006, Viscosol =  1.02 ±  0.005, ****p < 0.0001; Brain: STZ =  1.08 ±  0.01, Viscosol =  1.02 ±  0.01, ****p < 0.0001].

To evaluate the effect of Viscosolon cellular architecture, histological analysis of the kidney and brain tissues were performed. Kidney tissues of STZ group showed abnormal morphology with tubular dilation, tubular epithelial cell necrosis, degeneration, and vacuolation of renal tubules, tubular interstitial fibrosis, glomeruli shrinkage, thickening of bowman capsule, and mesangial matrix deposition. Whereas, in the brain tissues of the STZ group, the most obvious histological changes were increased axonal swelling, loss of axons and Schwann cells, and neuronal disorganization (Fig 2a). In the Viscosol treated group, partial retrieval towards normal morphology was observed in kidney and brain tissues. Cell count score was significantly higher in Viscosol treated group as compared to STZ group [Kidney: STZ =  0.76 ±  0.01, Viscosol =  0.84 ±  0.015, ****p < 0.0001); (Brain: STZ =  0.64 ±  0.01, Viscosol =  0.924 ±  0.01, ****p < 0.0001], indicating that Viscosolpartiallyrestored tissue damage and promoted cell recovery, as shown in (Fig 2b). Additionally, quantitative analysis also showed significant reduction ininflammatory lobules in the Viscosol treated group as compared to STZ group [Kidney: STZ =  1.15 ±  0.006, Viscosol =  1.02 ±  0.005, ****p < 0.0001; Brain: STZ =  1.08 ±  0.01, Viscosol =  1.02 ±  0.01, ****p < 0.0001] as shown in (Fig 2c).

### Gene expression profiling of inflammatory mediators involved in diabetic neuro and nephropathy

We further investigated the effect of Viscosol on the expression level of various T2D-induced inflammatory genes at the transcriptomic level through RT-qPCR by using *HPRT1* and *PPiA* as housekeeping genes in the kidney and brain tissues respectively.

#### Viscosol reduced the excess free fatty acids uptake and *de novo* lipogenesis.

**Fig 3:**
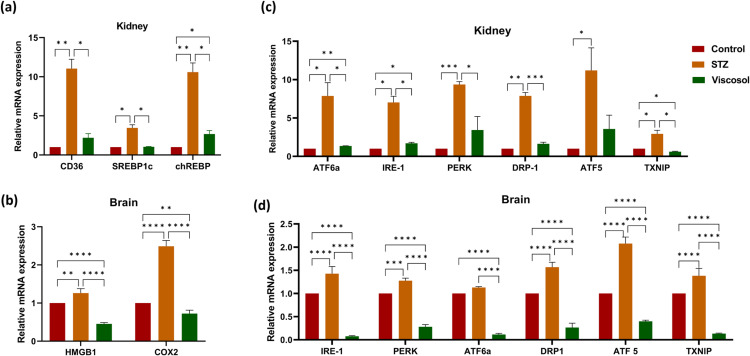
The expression profile of lipogenic genes, oxidative stress mediators as well as UPR^ER^ and mitochondrial stress markers. (a) Relative abundance of *CD36, chREBP*, and *SREBP1c* mRNA levels in diabetic mice group whose expression successfully reduced in the Viscosol treated group. (b)Relative expressionof*HMGB1* and *COX2* level, in all three groups, respectively. (c, d)Relative mRNA level of UPR^ER^ markers, i.e., *ATF-6a, PERK, IRE-1*, and mitochondrial stress markers, i.e., *DRP1*, *ATF5,* and *TXNIP* as the fold change activity in the Viscosol treated group, compared to the STZ group. Values are mean ±  SD, with their standard errors denoted by vertical bars. Ordinary two-way ANOVA followed by Tukey’s test was applied, and results were found to be significant. (****p < 0.0001, ***p < 0.0001, **p <  0.01 and * *p* < 0.05).

During the HFD state, lipid accumulates in the kidney tissues causing lipotoxicity that accelerates the production of oxidative stress, resulting in dyslipidemia and an inflammatory cascade. Therefore, we analyzed the relative mRNA expression of genes involved in lipid uptake (*CD36*) and *de novo* lipogenesis (*chREBP* and *SREBP1c*). We found significant results in all three groups (Control, STZ, Viscosol treated group) as depicted in (Fig 3a). We observed11-foldincreased relative mRNA expression of *CD36*and*chREBP*, whereas 3.5-fold increased expression of *SREBP1c*in the STZ group as compared to the control. However, Viscosol treatment reduced the expression of *CD36*, *chREBP* and *SREBP1c*upto 5.6-fold, 8.5-fold and 2.4-foldrespectively as compared to STZ group. Thus, it is suggested that Viscosol successfully protects visceral organs from diabetes-induced ectopic lipid accumulation and lipogenesis.

#### Viscosol decreased the inflammation and cellular oxidative stress.

In the diabetic milieu, hyperglycemia promotes the release of HMGB1 and COX2 proteins, which exacerbate ROS formation intensifying the inflammatory cascade. The relative mRNA expression of HMGB1 and COX-2 was significantly increased up to 1.4-fold and 2.3-fold in the brain tissues of the STZ group as shown in (Fig 3b).However, Viscosol reduced the expression to 1-fold and 1.6-fold respectively with respect to untreated STZ group.

#### Viscosol ameliorated the hyperactivated unfolded protein response (UPR^ER^) and mitochondrial stress.

UPR^ER^ mediators contribute to low-grade persistent chronic inflammation. An imbalance in mitochondrial dynamics also results in the onset of metabolic disturbances. Quantitative analysis showed increased expression of*IRE-1*, *PERK* and *ATF6a*in the STZ group, whereas treatment with Viscosol successfully downregulated their expression up to 6-fold,7-fold and 6-fold respectively in the kidney and 1-fold in brain tissues of Viscosol treated group, thus normalizing the UPR^ER^ response as shown in (Fig 3c,d). We further observed increased mRNA expression of mitochondrial fission protein DRP1, ROS-sensitive protein TXNIP, and ATF5, an apoptotic mitochondrial protein involved in UPR^mt^ in STZ group. However, Viscosol significantly downregulated their expression up to 6-fold, 3-fold and 8-foldin kidney tissues and 1.3-fold, 1.2-fold and 2-fold in brain tissues respectively, as compared to untreated STZ group.

#### Viscosol abolished the level of upstream mediators involved in inflammasome activation.

**Fig 4:**
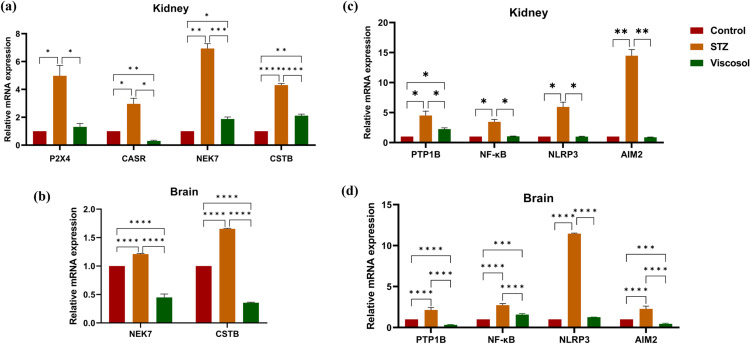
Effect of Viscosol on upstream mediators of inflammasomes activation, PTP1B and inflammasome complexes. (a, b)Relative mRNA level of *P2X4, CaSR, NEK7,* and *CSTB* in the kidney and brain samples. Increased expression was observedin the STZ group whereas reduced expression in the Viscosol treated group. (c, d)Relative mRNA expression of *PTP1B, NF-κB, NLRP3*, and *AIM2*.Viscosol treatment significantly reduced their expression as compared to STZ group. Ordinary two-way ANOVA and Tukey’s test was performed, and results were found to be significant. (Data represented as Mean ±  SD, ****p < 0.0001, ***p < 0.0001, **p < 0.01 and * *p* <  0.05).

Inflammasomes activation is one of the key factors in the pathogenesis of inflammation in diabetic neuro and nephropathy. Hence, we evaluated the relative mRNA expression of genes involved in ionic flux imbalance and disrupted lysosomal trafficking. The relative mRNA expression of *P2X4, CASR*, and *NEK7* as well as *CSTB* was found to be 4.9-fold, 2.9-fold, 6.5-fold and 4.1-fold enhanced in the STZ group of kidney tissues, while minimal expression was detected in the Viscosol treated group (Fig 4a). In brain tissues, the expression of NEK7 and CSTB was found to be 1.1-fold and 1.6-fold increase in STZ group and reduced after Viscosol treatment as shown in (Fig 4b). Our results suggested that the PTP1B inhibitor alleviates the expression of genes associated with activation stimuli for inflammasome complexes.

#### Viscosol mediated inhibition of PTP1B also reduced the hyperactivated NLRP3 and AIM2 inflammasomesexpression.

Inflammation is one of the crucial aspects in the pathogenesis of diabetic complications. PTP1B is not only an important negative regulator of the insulin signaling pathway but also a regulator of inflammatory cytokines, thus triggering the cascade of inflammatory responses. To explore the anti-inflammatory effect of Viscosol, the relative mRNA expression of *PTP1B*, *NF-κB*, and inflammasome complexes were evaluated. Quantitative analysis showed that Viscosol significantly reduced them RNA expression of *PTP1B, NF-κB, NLRP3*, and *AIM2*up to 2.7-folds, 2.6-folds, 5.7-folds and 14.2-folds in the kidney tissues (Fig 4c) and 1.6-folds, 1.2-folds, 10.2-folds and 1.5-folds in brain tissues as compared to STZ group as shown in (Fig 4d). Thus, suggesting that Viscosol has a potential inhibitory effect on PTP1B and shows anti-inflammatory activity by downregulating the *NF-κB*, inhibiting the transcription of NLRP3 and AIM2 inflammasomes.

#### Viscosol alleviated the inflammatory cytokines and chemokines level.

**Fig 5:**
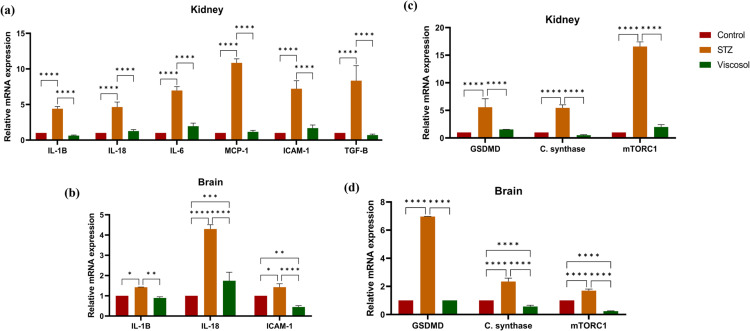
Effect of Viscosol on the expression of inflammatory cytokines, chemokines as well as cell death pathways. (a, b) Relative mRNA expression of inflammatory cytokines; *IL-1β, 1L-18,* and *IL-6* and chemokines; *MCP-1, ICAM-1,* and *TGF-β*. An increased trendline in the expression of respective markers was observed in STZ group, whereas decreased expression was observed in Viscosol treated group. (c, d) Relative mRNA expression of mediators of cell death pathways; pyroptosis, apoptosis, and autophagy.An increased expression of *GSDMD* and *cardiolipinsynthase*was observed in STZ group and reduced expression was observed in Viscosol treated group, respectively. Expression of *mTORC1* was elevated in the STZ group whereas reduced in the Viscosol treated group. The results shown are represented as mean ±  SD. Significant differences compared to control (****p < 0.0001, ***p < 0.0001, **p < 0.01 and * *p* <  0.05; two-wayANOVA and Tukey’s test).

Transcriptional regulation of cytokines and chemokines involved in regulating the inflammatory response were also analyzed. RT-qPCR analysis validated increased mRNA levels of inflammatory cytokines; *IL-1β*(4-fold), *IL-18*(4.8-fold),and *IL-6*(6-fold)as well as chemokines;*MCP-1*(10-fold), *ICAM1*(7-fold), and *TGF-β*(7-fold) in kidney tissues of STZ group. Viscosol treatment significantly reduced their expression as compared to STZ group as shown in (Fig 5a).In brain tissues, the expression of *IL-1β*(1.1-fold), *IL-18*(4-fold), ICAM1(1.3-fold)was significantly increased in STZ group. However, Viscosol treatment significantly reduced their expression as compared to disease as shown in (Fig 5b). These results suggested that Viscosol successfully ameliorated the expression of cytokines and chemokines, thus inhibiting the aggravation of inflammatory responses.

#### Viscosol inhibited the inflammatory cell death pathways and induced cytoprotective autophagy.

During diabetic complications, various inflammatory cell death pathways such as pyroptosis and apoptosis are activated, whereas autophagy is inhibited, which results in impairment in the clearance of toxic substances that leads to the accumulation of DAMPs that further primes NLRP3 and AIM2inflammasomes.Pyroptosis has been linked to NLRP3-Casp1 activation, escorted by GSDMD-mediated pores formation in plasma membranes. The presence of cardiolipin protein in the cytosol is an indication of apoptotic cell death, whereas mTORC1 is an inhibitor of autophagy. Weobservedsignificantly increased mRNA expression of *GSDMD,CLSand mTORC1*in STZ group. However, Viscosol treatment significantly reduced the expressionup to 4-fold, 2-fold, 14-fold in kidney tissues and 5-fold, 1-fold, 1.5-fold in brain tissues respectively as compared to STZ group.Thus,authenticating that Viscosol exhibited a protective effect against cell death mechanisms such as apoptosis and pyroptosis and induction of cytoprotective autophagy as shown in (Fig 5c, d).

### ADME/T analysis

**Fig 6:**
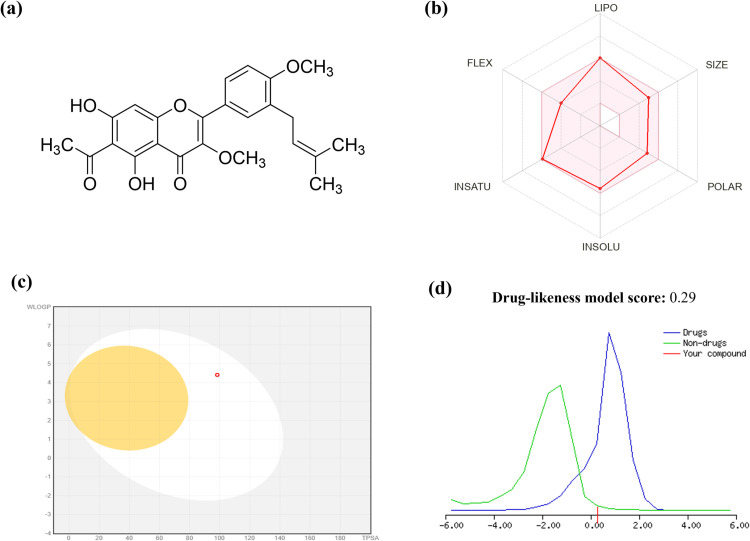
Viscosol structure and ADME/T profiling. (a) Chemical structure of Viscosol (b) Oral bioavailability radar, depicting Lipinski’s and Veber’s analysis. (c) BOILED-Egg model for Viscosol using SwissADME. The points in BOILED-yolk Egg’s region indicate the probability of molecules passively permeating the blood–brain barrier, whereas BOILED-white Egg’s region indicates the passive absorption by Gastrointestinal tract. (d) Drug-likeness score of Viscosol using MolSoft tool. Viscosol has a value of 0.29. The Non-drug-like behavior is denoted by (a green color curve), while drug-like behavior denoted by (a blue color curve). Compounds with a negative or zero value should not be considered drugs.

The basic physiochemical and pharmacokinetics properties as well as drug likeliness and toxicity model reports of Viscosol are presented in the Supplementary file (S2 Table). Lipinski’s rule of five demonstrates that on the basis of five parameters; molecular weight < 500 g/mol, hydrogen bond acceptor < 10, hydrogen bond donor < 5, lipophilicity (LogP) ≤ 5 and molar refractivity (TPSA) < 140 Å2, the biologically active molecule can likely be considered a promising orally bioavailable drug [23]. Viscosol obeyed Lipinski’s, Egan, Ghose, and Veber’s rules and exhibited no Blood-Brainbarrier (BBB) permeability and high GI absorption as demonstrated in (Fig 6b, c). MOLSOFT tool predicted the drug likeliness score (normal range −0.4 to 5.6) of 0.29 as shown in (Fig 6d). Viscosol successfully validated all rules with zero violation and the bioavailability score was found to be 0.55 (normal range 0 to 1) (S3 Table). Toxicological predictions computed by Pro-Tox II indicated that Viscosol is non-carcinogenic, non-hepatotoxic, and non-cytotoxic predicted (LD50 of 5000 mg/kg), making it a potential and safe drug candidate for clinical trials.

### Molecular docking analysis of Viscosol with PTP1B, NLRP3 and AIM2

**Fig 7:**
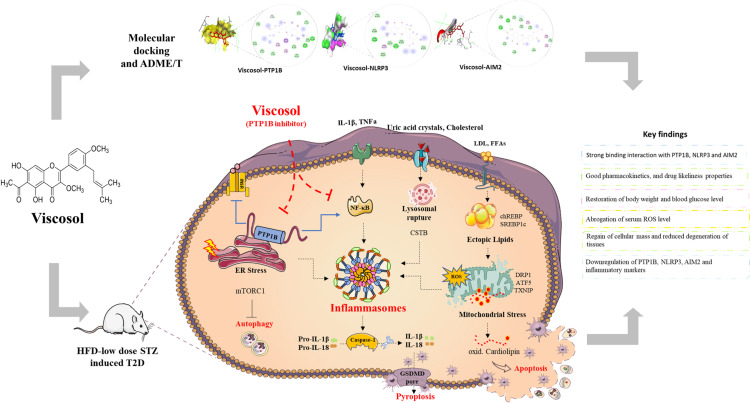
Schematic illustration of Viscosol mediated amelioration of meta-inflammation in T2D mice model.

**Fig 8:**
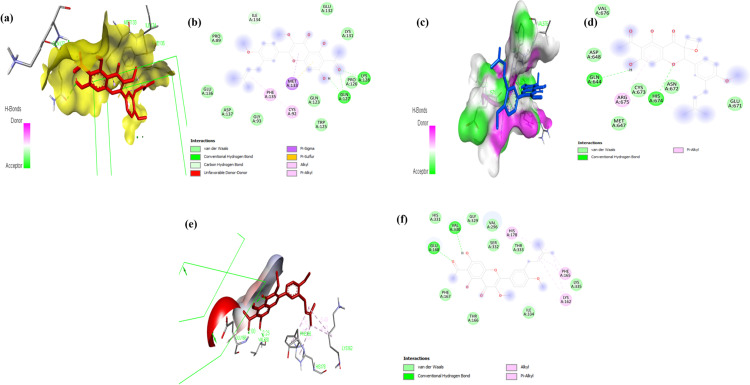
Representative 2D and 3D images ofmolecular docking of Viscosol. (a) 3D view of Viscosol in the binding pocket of PTP1B. (b) 2D configuration of interacting active amino acid residues of Viscosol with PTP1B (highlighting the H-bonding interaction with active amino acids GLU A:127; LYS A:128, and one pi-sigma bond through MET A: 133).(c)3D view of Viscosol in the binding pocket of NLRP3. (d)2D configuration of interacting active amino acid residues of Viscosol with NLRP3 (highlighting the H-bonding interaction withactive amino acids GLU A:644, HIS A:674). (e) 3D view of Viscosol in the binding pocket of AIM2. (f) 2D configuration of interacting active amino acid residues of Viscosol with AIM2 (highlighting the H-bonding interaction withactive amino acids GLU A:168, VAL A:330, LYS A:162).

Molecular docking has been demonstrated to be an effective method for accelerating the drug discovery process, besides being a potent tool for understanding various protein activities [24]. In the current study, we performed molecular docking to confirm the anti-inflammatory effects of Viscosol on NLRP3 and AIM2 inflammasome activation by investigating their potential binding modes (Fig 7). In order to better understand and elucidate the inhibitory activity of Viscosol as well as to explore its possible binding modes such as hydrogen bonding, alkyl, π -alkyl, π-sigma, π-π stacking, and Van der Waals interactions, the docking studies were carried out using the PyRx with DSV software. To assess the docked complexes, the minimum energy values (expressed as kcal/mol) and bonding interaction patterns were employed. The docking simulation for PTP1B was accomplished with the subsequent parameters: size (x =  20; size y =  20 and size z =  20), center (x =  19.0329; center y =  − 3.3137; center z =  16.751). The parameters and settings used in docking simulations with PyRx for NLRP3 are size (x =  20; size y =  20 and size z =  20), center (x =  11.0211; y =  − 2. 3451; z =  13.803). Similarly, For AIM2 the docking simulations are size (x =  20; size y =  20 and size z =  20), center (x =  15.1219; y =  − 3. 1352; z =  17.102). Auto dock 4.0 used Lamarckian algorithms. Default settings of the software were used for evaluating the optimal binding orientations and conformations of the ligand molecules to various protein inhibitors. The binding pattern of the Viscosol and ibuprofen, with PTP1B along with NLRP3 and AIM2 in 3D and 2D view are represented in (Fig 8a-f).

Docking outcomes revealed that Viscosol exhibits a good binding score with PTP1B having binding affinity (−6.8 kcal/mol) with active residue amino acids forming two hydrogen bonds like GLU A:127 LYS A:128, and one pi-sigma bond through MET A: 133 (Fig 8b). Similarly, Viscosol was also docked with NLRP3, having binding affinity (−6.4 kcal/mol), active residues forming hydrogen bonds with GLU A:644, HIS A:674 compared with Ibuprofen, binding affinity (−7.2 kcal/mol), taken as standard(Fig 8d). Furthermore, a significant interaction was observed between the Viscosol and amino acid residues of AIM2 having binding affinity of (−7.4 kcal/mol) through noncovalent interaction (Hydrogen bonding) like GLU A:168, VAL A:330, LYS A:162 as presented in (Fig 8f) (S4 Table).

## Discussion

Viscosol isolated from *Dodonaeaviscosa,* is a metabolically active prenylated flavonoid and a potent PTP1B inhibitor having IC_50_ of 13.5 μM and exhibits morefold inhibitory activity than other isolated compounds [14]. The antidiabetic potential of Viscosol has already been confirmed *in vivo*in our previous study. Viscosol improved insulin resistance by significantly up regulating INSR, IRS1, PI3K, andGLUT4 expression at both transcriptional and translational levels [15]. Current study aimed to investigate its pharmacological profile, binding interactions, antioxidant, and anti-inflammatory potential through *in silico* and *in vivo* studies. We developed a low-dose STZ and HFD-induced diabetic mouse model having pathological characteristics similar to the human diabetic phenotype [25].Previous studies have reported that PTP1B is a key negative regulator of insulin pathway leading to insulin resistance, hyperglycemia and dyslipidemia [26,27].Consistent with this report, our study also showed increased BGL and oxidative stress as well as reduced body weight of STZ group mice. However, treatment with Viscosol, a PTP1B inhibitor, successfully reversed diabetes induced metabolic dysregulations.

Furthermore, Viscosol also exhibited protective effects against cellular injury, tissue damage and inflammatory lobules in kidney and brain tissues. The histopathological analysis showed that kidney tissues of STZ group exhibited necrosis, fibrosis, thickening of bowman’s capsule, and mesangial matrix deposition in diabetic kidney tissuesconcurrent with the literature [28,29]. Furthermore, in the brain tissues of STZ group mice, axonal swelling, myelin vacuolation, and neuronal fiber disorganization was observedas previously reported [30].

Moreover, to investigate anti-inflammatory potential of Viscosol, we checked the relative mRNA expression of various regulators that exacerbate the inflammatory responses in the kidney and brain. Initially, we investigated the mRNA level of *PTP1B, NF-κB, NLRP3*, and *AIM2*.Viscosol treatment significantly reduced not only *PTP1B*expression but also decreased *NF-κB*expressionthus suppressing inflammation, parallel to previous studies [31,32]. We have observed an enhanced *NLRP3*and *AIM2* mRNA level in the STZ group, demonstrating a strong relation between T2Dand inflammasomes associated inflammation that has already been reported [33–35]. However, Viscosol treatment significantly reduced their expression. Currently, three acceptable models elucidate the possible mechanisms involved in the activation step of the NLRP3 inflammasome: K^ +^ efflux through P2X4 receptors [36], NEK7 protein [37], and increased Ca^2 +^ influx via calcium-sensing receptor CaSR [38]. Concordant with these studies, the expression of all respective markers was found upregulated in the STZ group and reduced expression was found in Viscosol treated group. Another hypothesis states that lysosomal perturbation and ROS also contribute to the NLRP3 activation via the release of CSTB [39] and activation of TXNIP protein [40] respectively. Like these findings, we have also found elevated *TXNIP* and *CSTB* levels in the STZ group and reduced expression in Viscosol treated group. Inflammasome complexes release proinflammatory cytokines, i.e., IL-1β and IL-18 that subsequently trigger a cascade of inflammatory responses [41]. Similarly, we also observed upregulated cytokines level in the kidney and brain tissues of the STZ group. Our results go in line with Mezzano *et al.* study that *NF-κB* plays a key role in the transcription of various proinflammatory chemokines such as*MCP-1* and *ICAM-1* that are involved in the progression of inflammatory responses [42]. To investigate the effect of Viscosol on lipotoxicity, we checked the expression of *CD36, SREBP1c*, and *chREBP,* whose expression increases in T2D [43,44]. Viscosol significantly decreased the expression of these markers. In our study, the mRNA expressions of UPR^ER^ players, i.e., *PERK, IRE-1,* and *ATF-6α,*were elevated in the STZ group whereas Viscosol mediated inhibition of PTP1B significantly downregulated their expression as well. Our findings are supported by a previous study that PTP1B deletion results in the downregulation of IRE-1 signaling, and PTP1B potentiates the PERK axis of UPR thus intensifying the inflammatory response [45]. ER stress mediators PERK and IRE-1 regulate the transcription of ATF5 (aUPR^mt^ protein) that potentiates inflammatory processes by activation of NLRP3 inflammasome [46]. These results were in line with our study. Elevated expression of *DRP1* accelerates oxidative stress and apoptosis in neurons of diabetic mice [47]. Our resultsare also consistent with previous studies. We found elevatedlevels of *COX2* and *HMGB1* in the STZ group [48]. Furthermore, Viscosol successfully decreased the *GSDMD* and cytochrome C level, that are major hallmarks of cell death [49].

According to ADME/T analysis [18], Viscosol obeyed Lipinski’s, Egan, Ghose, and Veber’s rules with zero violation, which indicates good absorption properties of Viscosol. MOLSOFT tool predicted the drug likeliness score (normal range −0.4 to 5.6) of 0.29. Toxicological predictions computed by Pro-Tox II indicated that Viscosol is non-carcinogenic, non-hepatotoxic, and non-cytotoxic, making Viscosol a potential drug candidate for clinical trials. For molecular docking studies, the interaction pattern of Viscosol and ibuprofen, with PTP1B along with NLRP3 and AIM2 was matched with previously published interaction patterns. The specific criteria of the binding pattern of the ligandswith the active siteswere taken from previous literature forPTP1B [50,51], NLRP3 [52] and AIM2 [53] respectively. Viscosol exhibited good binding energy and docking score with PTP1B, NLRP3 and AIM2. The chemical interactions such as (H bond, Van der Waals, and hydrophobic interacting residues), are comprehensively discussed in the results section.

## Conclusion

The present study validates that Viscosol exhibits outstanding pharmacological and therapeutic properties enumerated by computational investigation, *in silico* molecular docking as well as *in vivo* experiments. Computational profiling revealed good ADME properties, least toxicity, and high docking score of Viscosol with PTP1B, NLRP3, and AIM2. Our study substantiates that therapeutic administration of Viscosol successfully ameliorates ectopic lipid accumulation, oxidative damage, and inflammation in T2D mouse model. Overall, our results demonstrated that Viscosol exhibits anti-inflammatory activity by reversing the NLRP3 and AIM2-mediated inflammation in diabetic neuro and nephropathy, thus making it a potent therapeutic drug for the treatment of diabetes-linked pathophysiology.

## Supporting information

S1 Table Primers list for RT-qPCR.
(DOCX)

S2 Table Physicochemical Properties of Viscosol evaluated by Lipinski’s and Veber’s Rule
(DOCX)

S3 Table Predicted Pharmacokinetics, Toxicity, and Drug-likeness Profile of Viscosol
(DOCX)

S4 Table Binding affinity score of Viscosol to PTP1B, NLRP3, and AIM2
(DOCX)
