# Correction: Targeting NLRP3 and AIM2 signaling pathways by Viscosol alleviates metabolic dysregulations induced inflammatory responses in diabetic neuro- and nephropathy: An *in silico* and *in vivo* study

**DOI:** 10.1371/journal.pone.0338855

**Published:** 2025-12-11

**Authors:** Summan Thahiem, Muhammad Ihsan, Hamza Muneer, Aamir Sohail, Mehmand Khan, Iram Murtaza, Zia Uddin, Muhammad Shafique, Khalid J. Alzahrani, Hamid Ali, Imran Ullah

There is an error in affiliation 5 for author Muhammad Shafique. The correct affiliation 5 is: Department of Pharmaceutics, College of Pharmacy, Shaqra University, Shaqra-11961, Saudi Arabia.

In the Abstract section, there is a typographical error in the first sentence. The correct sentence is: Type 2 Diabetes (T2D) is a chronic metabolic disorder, considered the fastest growing pandemic of the 21^st^ century.

In the Abstract section, there is a typographical error in the fourth and fifth sentences. The correct sentences are: The concept of diabetes as an inflammatory disease has changed the pathogenic vision of T2D and hence, the compounds that mitigate inflammation in the setting of T2D are under the limelight of research. Current study aimed to evaluate the anti-inflammatory potency of Viscosol, a novel PTP1B inhibitor, isolated from Dodonaea viscosa, in the STZ-HFD-induced T2D mouse model.

In the Abstract section, there is a typographical error in the ninth sentence. The correct sentence is: The RT-qPCR analysis showed that Viscosol significantly reduced the mRNA expression of PTP1B, NF-κB, NLRP3, and *AIM2* up to 2.7-folds, 2.6-folds, 5.7-folds and 14.2-folds in the kidney tissues and 1.6-folds, 1.2-folds, 10.2-folds and 1.5-folds in brain tissues.

In the Introduction section, there is a typographical error in the third to fifth sentences of the second paragraph. The correct sentences are: They function as unique sensors for metabolic dysregulation. NLR Family, Pyrin Domain Containing 3(NLRP3) and Absent in Melanoma 2 (AIM2) inflammasomes are best known so far for their involvement in T2D [6,7].Several metabolic DAMPs such as (ROS, disrupted lysosomal trafficking, aberrant ionic flux, and cholesterol crystals) lead to assembly of inflammasome components, i.e., NLRP3/AIM2, apoptosis-associated speck-like protein containing a CARD (ASC), and caspase-1, subsequently releasing inflammatory cytokinesIL-1*β* and IL-18,and amplifying the inflammatory cascade [8]. It is worth emphasizing that inflammatory mediators IL-6 and Tumor Necrosis Factor α (TNF-α), lead to the activation of Protein Tyrosine Phosphatase 1B (PTP1B) [9,10].

In the Introduction section, there is a typographical error in the eighth sentence of the second paragraph. The correct sentence is: These properties render PTP1B an attractive target for therapeutic interventions.

In the Introduction section, there is a typographical error in the first and fifth sentences of the third paragraph. The correct first sentence is: Viscosol (5,7-dihydroxy-3,6- dimethoxy-2-(4-methoxy-3-(3- methyl but- 2-enyl) phenyl)- 4H-chromen-4-one) isolated from *Dodonaea viscosa* is a metabolically active naturally occurring prenylated flavonoid and potent PTP1B inhibitor [14]. The correct fifth sentence is: Furthermore, we investigated the putative association between the intricate molecular mechanisms linking PTP1B, NLRP3 and AIM2 inflammasomes as well as elucidating the anti-inflammatory potency of Viscosol *in vivo*.

In the Materials and methods section, there is a typographical error in the first, third and fifth sentences of the first paragraph. The correct first sentence is: Streptozotocin (STZ) was purchased from (Bioworld, Catalog No. # 41910012−3). The correct third sentence is: Invitrogen TRIzol reagent was purchased from Thermofisher Scientific. The correct fifth sentence is: Viscosol was sourced from Dr. Ziauddin, Assistant professor, at COMSATS University, who previously isolated this compound from aerial parts of *Dodonaea viscosa*.

In the Materials and methods section, there is a typographical error in the subheading Dose regimen for T2D model development is spelled incorrectly. The correct subheading is: Dose regimen for T2D model development.

In the Dose regimen for T2D model development subsection of the Materials and Methods, there is a typographical error in the first sentence. The correct sentence is: Male mice (Mus musculus, strain C57BL/6), weighing 25–40 g (8–12 weeks old) were selected as an experimental model.

In the Oxidative stress profiling using D-ROM assay subsection of the Materials and Methods, there is a typographical error in the third sentence. The correct sentence is: Both reagents (R1 and R2) were mixed well, maintaining the ratio of 1:24 to set up a homogeneous reaction mixture.

In the Molecular docking studies on PTP1B, NLRP3 and AIM2, there is a typographical error in the subheading Proteins and ligands preparation. The correct subheading is: Proteins and ligands preparation.

In the Molecular docking subsection of the Molecular docking studies on PTP1B, NLRP3 and AIM2, there is a typographical error in the second sentence. The correct sentence is: The results were visualized and evaluated using Discovery StudioVisualizer [22].

In the Results section, there is a typographical error in the heading Pharmacodynamics analysis. The correct heading is: Pharmacodynamics analysis.

In the caption of Fig 1, there is a typographical error in the second sentence. Please see the correct Fig 1 caption here.

**Fig 1. Baseline characteristics and oxidative profiling. (a)** Mean body weight (g) of all mice against experimental days. A significant regain in body weight was observed in the Viscosol treated group (****p < 0.0001). **(b)** Blood glucose profiling. The graph shows a decrease in blood glucose level in the presence of Viscosol (***p < 0.001). **(c)** ROS profiling. Viscosol treatment significantly reduced the ROS level (*p < 0.05).

In the Results, there is a typographical error in the heading Viscosol abolished diabetes-induced oxidative stress. The correct heading is: Viscosol abolished diabetes-induced oxidative stress.

In the Viscosol abolished diabetes-induced oxidative stress subsection of the Results, there is a typographical error in the third sentence of the paragraph. The correct sentence is: In the Viscosol treated group, there was a significant abrogation in serum ROS level as compared to the STZ group(***p < 0.0531).

In the Protective effect of Viscosol on nephro- and neuro histopathology subsection of the Results, there is a typographical error in the first, fifth to sixth sentences of the paragraph. The correct first sentence is: To evaluate the effect of Viscosol on cellular architecture, histological analysis of the kidney and brain tissues were performed. The correct fifth and sixth sentences are: Cell count score was significantly higher in Viscosol treated group as compared to STZ group [Kidney: STZ = 0.76 ± 0.01, Viscosol = 0.84 ± 0.015, ****p < 0.0001); (Brain: STZ = 0.64 ± 0.01, Viscosol = 0.924 ± 0.01, ****p < 0.0001], indicating that Viscosol partially restored tissue damage and promoted cell recovery, as shown in (Fig 2b). Additionally, quantitative analysis also showed significant reduction in inflammatory lobules in the Viscosol treated group as compared to STZ group [Kidney: STZ = 1.15 ± 0.006, Viscosol = 1.02 ± 0.005, ****p < 0.0001; Brain: STZ = 1.08 ± 0.01, Viscosol = 1.02 ± 0.01, ****p < 0.0001] as shown in (Fig 2c).

In the caption of Fig 2, there is a typographical error in the second sentence. Please see the correct Fig 2 caption here.

**Fig 2. Photomicrographs of H & E-stained kidney and brain section at 10X. (a)** Normal morphology of the kidney tissue; STZ group, presenting histological changes; tubular dilation (*), epithelial cell necrosis (α), degeneration of renal tubules (β), tubular interstitial fibrosis (box), glomeruli shrinkage and thickening of bowman capsule (ε) mesangial matrix deposition (γ); partial retrieval towards normal morphology in Viscosol treated group. The second panel shows brain normal tissues; HFD-STZ group, presenting altered tissue morphology having increased axonal swelling (arrowhead), loss of axons (neon arrows), lost myelin sheath (ε), loss of Schwann cells (arrow), and neuronal disorganization (*); Viscosol treated group, showing partial retrieval towards normal morphology. **(b)** Cell count score was significantly higher in Viscosol treated group as compared to STZ group [Kidney: STZ = 0.76 ± 0.01, Viscosol = 0.84 ± 0.015, ****p < 0.0001); (Brain: STZ = 0.64 ± 0.01, Viscosol = 0.924 ± 0.01, ****p < 0.0001]. **(c)** Inflammatory lobules score showed significant reduction in inflammatory lobules in the Viscosol treated grou*p* as com*p*ared to STZ group [Kidney: STZ = 1.15 ± 0.006, Viscosol = 1.02 ± 0.005, ****p < 0.0001; Brain: STZ = 1.08 ± 0.01, Viscosol = 1.02 ± 0.01, ****p < 0.0001].

In the Viscosol reduced the excess free fatty acids uptake and de novo lipogenesis subheading of the Gene expression profiling of inflammatory mediators involved in diabetic neuro and nephropathy, there are typographical errors in the fourth to fifth sentences. The correct sentences are: We observed11- fold increased relative mRNA expression of *CD36* and *chREBP*, whereas 3.5-fold increased expression of *SREBP1c* in the STZ group as compared to the control. However, Viscosol treatment reduced the expression of *CD36*, *chREBP* and *SREBP1c* up to 5.6-fold, 8.5-fold and 2.4-fold respectively as compared to STZ group.

In the caption of Fig 3, there is a typographical error in the caption for panel B. Please see the correct Fig 2 caption here.

**Fig 3. The expression profile of lipogenic genes, oxidative stress mediators as well as UPRER and mitochondrial stress markers. (a)** Relative abundance of *CD36, chREBP*, and *SREBP1c* mRNA levels in diabetic mice group whose expression successfully reduced in the Viscosol treated group. **(b)**Relative expression of *HMGB1* and *COX2* level, in all three groups, respectively. **(c, d)**Relative mRNA level of UPRER markers, i.e., *ATF-6a, PERK, IRE-1*, and mitochondrial stress markers, i.e., *DRP1*, *ATF5,* and *TXNIP* as the fold change activity in the Viscosol treated group, compared to the STZ group. Values are mean ± SD, with their standard errors denoted by vertical bars. Ordinary two-way ANOVA followed by Tukey’s test was applied, and results were found to be significant. (****p < 0.0001, ***p < 0.0001, **p < 0.01 and * *p* < 0.05).

In the Viscosol ameliorated the hyperactivated unfolded protein response (UPR^ER^) and mitochondrial stress subsection of the Gene expression profiling of inflammatory mediators involved in diabetic

neuro and nephropathy subsection of the Results, there is an error in the third sentence. The correct sentence is: Quantitative analysis showed increased expression of *IRE-1*, *PERK* and *ATF6a* in the STZ group, whereas treatment with Viscosol successfully downregulated their expression up to 6-fold,7-fold and 6-fold respectively in the kidney and 1-fold in brain tissues of Viscosol treated group, thus normalizing the UPRER response as shown in (Fig 3c,d).

In the Results section, there is a typographical error in the subheading Viscosol mediated inhibition of PTP1B also reduced the hyperactivated NLRP3 and AIM2 inflammasomes expression. The correct subheading is: Viscosol mediated inhibition of PTP1B also reduced the hyperactivated NLRP3 and AIM2 inflammasomes expression.

In the Viscosol mediated inhibition of PTP1B also reduced the hyperactivated NLRP3 and AIM2 inflammasomes expression subsection of the Results, there is a typographical error in the fourth sentence. The correct sentence is: Quantitative analysis showed that Viscosol significantly reduced them RNA expression of *PTP1B, NF-κB, NLRP3*, and *AIM2* up to 2.7-folds, 2.6-folds, 5.7-folds and 14.2-folds in the kidney tissues (Fig 4c) and 1.6-folds, 1.2-folds, 10.2-folds and 1.5-folds in brain tissues as compared to STZ group as shown in (Fig 4d).

In the Viscosol alleviated the inflammatory cytokines and chemokines level subsection of the Results, there are typographical errors in the second and third sentences. The correct sentences are: RT-qPCR analysis validated increased mRNA levels of inflammatory cytokines; *IL-1β* (4-fold), *IL-18* (4.8-fold), and *IL-6* (6-fold) as well as chemokines; *MCP-1* (10- fold), *ICAM1*(7-fold), and *TGF-β* (7-fold) in kidney tissues of STZ group. Viscosol treatment significantly reduced their expression as compared to STZ group as shown in (Fig 5a). In brain tissues, the expression of *IL-1β* (1.1-fold), *IL-18* (4-fold), ICAM1 (1.3-fold) was significantly increased in STZ group.

In the caption of Fig 4, there is a typographical error in the caption for panel A and B. Please see the correct Fig 4 caption here.

**Fig 4. Effect of Viscosol on upstream mediators of inflammasomes activation, PTP1B and inflammasome complexes. (a, b)Relative mRNA level of P2X4, CaSR,** NEK7, and CSTB in the kidney and brain samples. Increased expression was observed in the STZ group whereas reduced expression in the Viscosol treated group. (c, d)Relative mRNA expression of PTP1B, NF-κB, NLRP3, and AIM2.Viscosol treatment significantly reduced their expression as compared to STZ group. Ordinary two-way ANOVA and Tukey’s test was performed, and results were found to be significant. (Data represented as Mean ± SD, ****p < 0.0001, ***p < 0.0001, **p < 0.01 and * p < 0.05).

In the Viscosol inhibited the inflammatory cell death pathways and induced cytoprotective

Autophagy subsection of the Results, there are typographical errors in the first, fourth to sixth sentences. The correct first sentence is: During diabetic complications, various inflammatory cell death pathways such as pyroptosis and apoptosis are activated, whereas autophagy is inhibited, which results in impairment in the clearance of toxic substances that leads to the accumulation of DAMPs that further primes NLRP3 and AIM2 inflammasomes. The correct fourth sentence is: We observed significantly increased mRNA expression of *GSDMD, CLS and mTORC1*in STZ group. The correct fifth sentence is: However, Viscosol treatment significantly reduced the expression up to 4-fold, 2-fold, 14-fold in kidney tissues and 5-fold, 1-fold, 1.5- fold in brain tissues respectively as compared to STZ group. The correct sixth sentence is: Thus, authenticating that Viscosol exhibited a protective effect against cell death mechanisms such as apoptosis and pyroptosis and induction of cytoprotective autophagy as shown in (Fig 5c, d).

In the caption of Fig 5, there are typographical errors in the caption for panel C and D. Please see the correct Fig 5 caption here.

**Fig 5. Effect of Viscosol on the expression of inflammatory cytokines, chemokines as well as cell death pathways. (a, b)** Relative mRNA expression of inflammatory cytokines; *IL-1β, 1L-18,* and *IL-6* and chemokines; *MCP-1, ICAM-1,* and *TGF-β*. An increased trendline in the expression of respective markers was observed in STZ group, whereas decreased expression was observed in Viscosol treated group. **(c, d)** Relative mRNA expression of mediators of cell death pathways; pyroptosis, apoptosis, and autophagy.An increased expression of *GSDMD* and *cardiolipinsynthase* was observed in STZ group and reduced expression was observed in Viscosol treated group, respectively. Expression of *mTORC1* was elevated in the STZ group whereas reduced in the Viscosol treated group. The results shown are represented as mean ± SD. Significant differences compared to control (****p < 0.0001, ***p < 0.0001, **p < 0.01 and * *p* < 0.05; two-way ANOVA and Tukey’s test).

In the ADME/T analysis subsection of the Results, there is a typographical error in the third sentence. The correct sentence is: Viscosol obeyed Lipinski’s, Egan, Ghose, and Veber’s rules and exhibited no Blood-Brain barrier (BBB) permeability and high GI absorption as demonstrated in (Fig 6b, c).

In the Discussion section, there is a typographical error in the first to third sentences of the first paragraph. The correct sentences are: Viscosol isolated from *Dodonaea viscosa,* is a metabolically active prenylated flavonoid and a potent PTP1B inhibitor having IC_50_ of 13.5 μM and exhibits morefold inhibitory activity than other isolated compounds [14]. The antidiabetic potential of Viscosol has already been confirmed *in vivo* in our previous study. Viscosol improved insulin resistance by significantly up regulating INSR, IRS1, PI3K, and GLUT4 expression at both transcriptional and translational levels [15].

In the Discussion section, there is a typographical error in the second and third sentences of the second paragraph. The correct sentences are: The histopathological analysis showed that kidney tissues of STZ group exhibited necrosis, fibrosis, thickening of bowman’s capsule, and mesangial matrix deposition in diabetic kidney tissues concurrent with the literature [28,29]. Furthermore, in the brain tissues of STZ group mice, axonal swelling, myelin vacuolation, and neuronal fiber disorganization was observed as previously reported [30].

In the caption of Fig 8, there are typographical errors in the caption for panel D and E. Please see the correct Fig 8 caption here.

**Fig 8. Representative 2D and 3D images ofmolecular docking of Viscosol. (a)** 3D view of Viscosol in the binding pocket of PTP1B. **(b)** 2D configuration of interacting active amino acid residues of Viscosol with PTP1B (highlighting the H-bonding interaction with active amino acids GLU A:127; LYS A:128, and one pi-sigma bond through MET A: 133).(c)3D view of Viscosol in the binding pocket of NLRP3. **(d)**2D configuration of interacting active amino acid residues of Viscosol with NLRP3 (highlighting the H-bonding interaction with active amino acids GLU A:644, HIS A:674). **(e)** 3D view of Viscosol in the binding pocket of AIM2. **(f)** 2D configuration of interacting active amino acid residues of Viscosol with AIM2 (highlighting the H-bonding interaction with active amino acids GLU A:168, VAL A:330, LYS A:162).

In the Discussion section, there are errors in the third to fourth, tenth, 13^th^, 15^th^, 17^th^ to 18^th^ sentences of the third paragraph. The correct third to fourth sentences are: Viscosol treatment significantly reduced not only *PTP1B*expression but also decreased *NF-κB* expression thus suppressing inflammation, parallel to previous studies [31,32]. We have observed an enhanced *NLRP3* and *AIM2* mRNA level in the STZ group, demonstrating a strong relation between T2D and inflammasomes associated inflammation that has already been reported [33–35]. The correct tenth sentence is: Our results go in line with Mezzano *et al.* study that *NF-κB* plays a key role in the transcription of various proinflammatory chemokines such as *MCP-1* and *ICAM-1* that are involved in the progression of inflammatory responses [42]. The correct 13^th^ sentence is: In our study, the mRNA expressions of UPRER players, i.e., *PERK, IRE-1,* and *ATF-6α,* were elevated in the STZ group whereas Viscosol mediated inhibition of PTP1B significantly downregulated their expression as well. The correct 15^th^ sentence is: ER stress mediators PERK and IRE-1 regulate the transcription of ATF5 (a UPRmt protein) that potentiates inflammatory processes by activation of NLRP3 inflammasome [46]. The correct 17^th^ sentence is: Our results are also consistent with previous studies. The correct 18^th^ sentence is: We found elevated levels of *COX2* and *HMGB1* in the STZ group [48].

In the Discussion section, there is an error in the fifth sentence of the fourth paragraph. The correct sentence is: The specific criteria of the binding pattern of the ligands with the active sites were taken from previous literature for PTP1B [50,51], NLRP3 [52] and AIM2 [53] respectively.

The Acknowledgement section was formatted incorrectly. The correct Acknowledgement section is: The authors would like to thank the Deanship for Scientific Research at Shaqra University for supporting the work.
