# Expression of Concern: Targeting NLRP3 and AIM2 signaling pathways by Viscosol alleviates metabolic dysregulations induced inflammatory responses in diabetic neuro- and nephropathy: An *in silico* and *in vivo* study

**DOI:** 10.1371/journal.pone.0342732

**Published:** 2026-02-12

**Authors:** The PLOS One Editors

After this article [1,2] was published, concerns were raised that the article cited as Reference 9 was retracted before publication of [1,2]. Additionally, the *PLOS One* Editors have concerns about the article’s compliance with the PLOS Authorship policy.

In light of the cumulative issues, the *PLOS One* Editors issue this Expression of Concern. Readers are advised to interpret the article [1,2] with caution.
