# Supplementary material for: Targeting NLRP3 and AIM2 signaling pathways by Viscosol alleviates metabolic dysregulations induced inflammatory responses in diabetic neuro- and nephropathy: An in silico and in vivo study
Source: PLoS One. 2025 Apr 2;20(4):e0313816. doi: 10.1371/journal.pone.0313816 (PMC11964203; doi:10.1371/journal.pone.0313816)
Supplement: S1 Table — (DOCX) [file pone.0313816.s001.docx]

**Table S1.** Primers list for RT-qPCR.

| **Primers** | **Accession number** | **5' to 3' sequence** | **TM (°C)** | **Amplicon size**  **(bp)** |
| --- | --- | --- | --- | --- |
| **mTxnip** | [NM_023719.2](https://www.ncbi.nlm.nih.gov/entrez/viewer.fcgi?db=nucleotide&id=254553443) | **F**: TTCCTGTCCAGTGTTGGGA  **R**: CTGCACAGTTCTCAGGTGGA | 60. 1 | 115 |
|  |  |  | 60. 0 |  |
| **mAtf-6α** | NM_001081304.1 | **F:** TTAGAGTGCCCGAAGCCA **R:** CCGATCTTCCCACTTCCAC | 60.5 | 99 |
|  |  |  | 60.5 |  |
| **mCstb** | NM_007798.3 | **F:**CTCTCCACCGCTCAAACACT  **R:** AGCGTGGTTAGTTGGCTACATT | 60.4 | 80 |
|  |  |  | 60.1 |  |
| **mGsdmd** | NM_026960.4 | **F**: CCCTTCCCACAACATCTCC  **R:** CTTGGCTTCCCAAAGGCT | 60.3 | 77 |
|  |  |  | 60.3 |  |
| **mP2rx4** | NM_011026.3 | **F:**TCCCAACAGCGGCATAAG  **R:** CAGAGGCGGCATGTTGTTA | 60.8 | 79 |
|  |  |  | 60.8 |  |
| **mIl1b** | NM_008361.4 | **F:** AGGGGACATTAGGCAGCAC **R:** AGTGCGGGCTATGACCAA | 60.1 | 78 |
|  |  |  | 60.2 |  |
| **mNf-kb 1** | NM_008689.2 | **F**: GCGTCCTTTCTTGGTTCTGA  **R**: GCTCAAGACACTGCACCTGA | 60. 4 | 114 |
|  |  |  | 60. 2 |  |
| **mIcam1** | NM_010493.3 | **F:** ATCACATGGGTCGAGGGTT **R:** GGATGTGGAGGAGCAGAGAA | 60. 2 | 119 |
|  |  |  | 60. 3 |  |
| **mIl6** | NM_001314054.1 | **F:**CGGCAAACCTAGTGCGTTAT  **R**: TCTGACCACAGTGAGGAATGTC | 60.2 | 63 |
|  |  |  | 60.2 |  |
| **mErn1** | NM_023913.2 | **F:** GCAGCCTTATCCACACTGCT  **R**: AACACACAGGGGAACAGGAG | 60. 4 | 64 |
|  |  |  | 60. 0 |  |
| **mDnm1l** | NM_001025947.2 | **F:**TCAGAAAGGAAGCTCCAGGA  **R**: GTGAGGAACTTGCCAGGCT | 60. 1 | 98 |
|  |  |  | 60. 4 |  |
| **mmTorc1** | NM_020009 | **F:** CCAGGAGGACATTTGTTCAGA  **R**:CACTGAACACAGTAGAGCCAGTG | 60.1 | 96 |
|  |  |  | 60.0 |  |
| **mNek7** | NM_001311148.1 | **F:** GTGCGCTGCTACCAGAATCT  **R:** GGGCAGTGGTCCTCACATAG | 60.6 | 68 |
|  |  |  | 60.5 |  |
| **mCasr** | NM_013803.3 | **F:**ACCACCAGCTCGGATGAA  **R**: AGAGGGAAGCAGGTGGGTAG | 60. 2 | 52 |
|  |  |  | 60. 6 |  |
| **mCrls1** | NM_001024385.1 | **F:**CAGATCCAGCAGCAAGGAA  **R**: TTCGCAAGCTGAACACCA | 60. 1 | 91 |
|  |  |  | 60. 1 |  |
| **mAtf5** | NM_030693.2 | **F:**CTGGGTGATGGCAGATGAG  **R**: CCTGGCTCCCTTTTCTTGA | 60. 2 | 97 |
|  |  |  | 60. 3 |  |
| **mTgf-b1** | NM_011577.2 | **F:**GGAGAGCCCTGGATACCAA  **R**: ACTTCCAACCCAGGTCCTTC | 60. 0 | 99 |
|  |  |  | 60. 3 |  |
| **mSrebp1c** | NM_011480 | **F:** GGACCTTTGTCATTGGCTGT  **R**: GCATGGTCCTGATTGCTTG | 60.0 | 96 |
|  |  |  | 60.2 |  |
| **mCd36** | NM_001159556 | **F:**TACAGAAGACCTGGGCTTGG | 60.2 | 104 |
|  |  | **R:**CAGAAGGGTGCACAGGAGA | 60.0 |  |
| **mCox2** | NM_011198.4 | **F:** GACTTGCCAGGCTGAACTTC | 60. 0 | 96 |
|  |  | **R:** GCTCACGAGGCCACTGATA | 60.0 |  |
| **mMlxipl** | NM_021455 | **F:** TGTCGGTCTGTTTCCTCACA  **R:** CTGCCTCTCTGCTCAGGAAC | 60.3 | 73 |
|  |  |  | 60.3 |  |
| **mHmgb1** | NM_010439.4 | **F:** CCATTTTGGGTCACATGGA  **R:** CAATGGCAGGGCATGTG | 60.2 | 85 |
|  |  |  | 60.7 |  |
| **mAim2** | [NM_001013779.2](https://www.ncbi.nlm.nih.gov/entrez/viewer.fcgi?db=nucleotide&id=162461984) | **F**: ACAATGGAGGTGTTGGTGCT  **R**: ATCTTGTCTCCTTCCTCGCA | 60.4 | 62 |
|  |  |  | 60.0 |  |
| **mNlrp3** | NM_001359638.1 | **F:**  AGCCCTCCTTCACCATCAG  **R:**  CACAAGCCTTTGCTCCAGA | 60.2 | 68 |
|  |  |  | 60.1 |  |
| **mEif2ak3** | NM_001313918.1 | **F:** ACTTCAAGGAAAGGGCTGTGT  **R:** AGTCTTGGGACACCGACAAG | 60.2 | 62 |
|  |  |  | 60.2 |  |
| **mIl18** | NM_001357221.1 | **F:** GGGAGGGTTTGTGTTCCAG  **R:** GCAGCCTCGGGTATTCTGT | 60.3 | 90 |
|  |  |  | 60.2 |  |
| **mHprt1** | NM_013556 | **F**: GGACAGGACTGAAAGACTTGCT  **R**: TGTAATCCAGCAGGTCAGCA | 59.9 | 116 |
|  |  |  | 60.4 |  |
| **mCcl1** | NM_011329.3 | **F:** AGGTCCTTGTCCGTGTCTTCT  **R:** TAGTTGAGGCGCAGCTTTCT | 60.2 | 70 |
|  |  |  | 60.3 |  |
| **mPpia** | [NM_008907.2](https://www.ncbi.nlm.nih.gov/entrez/viewer.fcgi?db=nucleotide&id=1418589268) | **F**: TTGGTCCGAAGTAGCCACA  **R:** GCCAAGCCTTTCTCGTTTC | 60.2 | 88 |
|  |  |  | 59.9 |  |
| **mPtp1n** | NM_001013779.2 | **F:** GCATAGGACAGTGGTAATGCG  **R:** AACTCACAGGGAAAGCAGAGG | 60.5 | 123 |
|  |  |  | 60.8 |  |
