# Supplementary material for: Targeting NLRP3 and AIM2 signaling pathways by Viscosol alleviates metabolic dysregulations induced inflammatory responses in diabetic neuro- and nephropathy: An in silico and in vivo study
Source: PLoS One. 2025 Apr 2;20(4):e0313816. doi: 10.1371/journal.pone.0313816 (PMC11964203; doi:10.1371/journal.pone.0313816)
Supplement: S2 Table — (DOCX) [file pone.0313816.s002.docx]

**Table S2.** Physicochemical Properties of Viscosol evaluated by Lipinski’s and Veber’s Rule

| **Physiochemical properties** | **Standard Value** | **Viscosol** |
| --- | --- | --- |
| Molecular weight | <500 g/mol | 412.43 g/mol |
| Lipophilicity | LogP ≤5 | 3.93 |
| No. of H-bond acceptor | <10 | 7 |
| No. of H-bond donor | <10 | 2 |
| Polar Surface area | TPSA<140 Å2 | 98.36 Å² |
| No. of rotatable bonds | <10 | 6 |
