# Supplementary material for: Targeting NLRP3 and AIM2 signaling pathways by Viscosol alleviates metabolic dysregulations induced inflammatory responses in diabetic neuro- and nephropathy: An in silico and in vivo study
Source: PLoS One. 2025 Apr 2;20(4):e0313816. doi: 10.1371/journal.pone.0313816 (PMC11964203; doi:10.1371/journal.pone.0313816)
Supplement: S3 Table — (DOCX) [file pone.0313816.s003.docx]

**Table S3.** Predicted Pharmacokinetics, Toxicity, and Drug-likeness Profile of Viscosol

| **Pharmacokinetics** | | **Toxicity Model Report** | |
| --- | --- | --- | --- |
| GI absorption | High | Hepatotoxicity | None |
| BBB permeability | No | Carcinogenicity | None |
| CYP1A2 inhibitor | No | Mutagenicity | None |
| CYP2C19 inhibitor | No | Cytotoxicity | None |
| CYP3A4 inhibitor | No | Phosphoprotein (Tumor Suppressor) p53 stress response pathway | None |
| Log *K*_p_ (skin permeation) | -5.22 cm/s | HSE stress response pathway | None |
