# Supplementary material for: Targeting NLRP3 and AIM2 signaling pathways by Viscosol alleviates metabolic dysregulations induced inflammatory responses in diabetic neuro- and nephropathy: An in silico and in vivo study
Source: PLoS One. 2025 Apr 2;20(4):e0313816. doi: 10.1371/journal.pone.0313816 (PMC11964203; doi:10.1371/journal.pone.0313816)
Supplement: S4 Table — (DOCX) [file pone.0313816.s004.docx]

**Table S4.**Binding affinity score of Viscosol to PTP1B, NLRP3, and AIM2

|  | **Binding Affinity Score (Kcal/mol)** | | |
| --- | --- | --- | --- |
| **Compounds** | **PTP1B** | **NLRP3** | **AIM2** |
| Viscosol | -6.8 | -6.4 | -7.4 |
| Ibuprofen | -7.2 | -5.5 | -6.8 |
